# Twenty Years of Modern Rectal Cancer Care in Lithuania: Stage Migration, Evolving Treatment Patterns, and Improved Survival at a Tertiary Referral Centre

**DOI:** 10.3390/medicina62101888

**Published:** 2026-09-29

**Authors:** Rita Ambraziene, Laura Kairevice, Lina Puzauskiene, Greta Chlebopaseviene, Algimantas Tamelis, Tadas Latkauskas, Henrikas Pauzas, Irina Gineikiene, Ingrida Pikuniene, Laimonas Jarusevicius, Vita Simonyte-Verbickiene, Dainius Janciauskas, Egle Butnoriute, Emilija Jaceviciute, Rasa Janciauskiene

**Affiliations:** 1 Department of Oncology and Hematology, Institute of Oncology, Lithuanian University of Health Sciences, 50161 Kaunas, Lithuania; laura.kairevice@kaunoklinikos.lt (L.K.); lina.puzauskiene@kaunoklinikos.lt (L.P.); greta_zindziute@yahoo.com (G.C.); laimonas.jarusevicius@kaunoklinikos.lt (L.J.); vita.simonyte-verbickiene@kaunoklinikos.lt (V.S.-V.); rasa.janciauskiene@kaunoklinikos.lt (R.J.); 2 Department of Oncology and Hematology, Hospital of Lithuanian University of Health Sciences, Kaunas Clinics, LT-50161 Kaunas, Lithuania; 3 Department of Surgery, Medical Academy, Lithuanian University of Health Sciences, 44307 Kaunas, Lithuania; algimantas.tamelis@kaunoklinikos.lt (A.T.); tadas.latkauskas@kaunoklinikos.lt (T.L.); henrikas.pauzas@kaunoklinikos.lt (H.P.); 4 Department of Radiology, Medical Academy, Lithuanian University of Health Sciences, 44307 Kaunas, Lithuania; irina.gineikiene@gmail.com (I.G.); ingrida.pikuniene@lsmu.lt (I.P.); 5 Department of Pathology, Medical Academy, Lithuanian University of Health Sciences, 44307 Kaunas, Lithuania; dainius.janciauskas@kaunoklinikos.lt; 6 Faculty of Medicine, Lithuanian University of Health Sciences, 44307 Kaunas, Lithuania; eglebutnor@gmail.com (E.B.); emilija.jaceviciute@kaunoklinikos.lt (E.J.)

**Keywords:** rectal cancer, overall survival, cancer-specific survival, neoadjuvant therapy, total neoadjuvant treatment, stage migration, real-world evidence, clinical implementation, colorectal cancer screening

## Abstract

*Background and Objectives*: Rectal cancer management has changed substantially over the past two decades. We evaluated 20-year trends in stage at diagnosis, treatment patterns, and survival at a Lithuanian tertiary referral centre. *Materials and Methods*: This retrospective cohort study included 839 patients who underwent resection for stage I–III rectal cancer between 2004 and 2023, divided into two periods: 2004–2013 (*n* = 426) and 2014–2023 (*n* = 413). Survival was analysed with Kaplan–Meier estimates and Cox proportional hazards regression, truncated at 60 months. *Results*: Patients in the later cohort were diagnosed at an earlier clinical stage (stage I: 12.6% vs. 2.4%) and more often received neoadjuvant or perioperative therapy (55.2% vs. 37.1%; *p* < 0.001). Among clinical stage III patients, receipt of neoadjuvant chemoradiotherapy or total neoadjuvant treatment rose from 15.9% to 70.9% (*p* < 0.001), while pathological complete response among treated patients remained stable (10.8% vs. 10.7%; *p* = 0.98). Five-year overall survival increased from 64.8% to 71.3% (HR 0.77; 95% CI 0.60–0.99; *p* = 0.039) and cancer-specific survival from 70.4% to 80.8% (HR 0.60; 95% CI 0.45–0.80; *p* = 0.0005). The cohort effect persisted after multivariable adjustment for CSS (HR 0.62; 95% CI 0.45–0.85; *p* = 0.003), with clinical stage explaining only ~13% of the crude effect; stage III sensitivity analysis confirmed the CSS gain (67.9% to 77.3%; *p* = 0.009). *Conclusions*: Cancer-specific survival improved substantially and independently of stage migration. The gain coincided with markedly wider and better-targeted delivery of effective neoadjuvant therapy rather than with improved per-patient tumour responsiveness. As an observational comparison of two historical cohorts, this study demonstrates association rather than causation; nonetheless, it provides real-world evidence consistent with the hypothesis that systematic implementation of evidence-based rectal cancer pathways translates into measurable survival benefit.

## 1. Introduction

Colorectal cancer is a major public health burden in high-income countries owing to its high prevalence and associated mortality [1]. In Europe, it is the second most common malignancy, with approximately 520,000 new cases and 250,000 deaths in 2020, accounting for 12.9% of all cancer diagnoses and 12.6% of cancer-related deaths [2]. Rectal cancer represents approximately 35% of colorectal cancer cases in the European Union, corresponding to roughly 125,000 new diagnoses annually, a figure projected to increase further [3].

Colorectal cancer survival has improved globally over recent decades, driven by advances in diagnosis and treatment [4]. However, substantial geographic disparities persist. Between 2010 and 2014, 5-year net survival for rectal cancer ranged from 60–69% in many Western and Northern European countries to 50–59% in the Baltic states, the Czech Republic, Malta, and Romania, and below 50% in Slovakia, Poland, Croatia, and Bulgaria [5]. Survival is strongly stage-dependent, reaching approximately 90% for stage I and just above 10% for stage IV disease [6]. These disparities may partly reflect variations in diagnostic and treatment practices at both national and institutional levels.

Over the past four decades, rectal cancer management has evolved substantially through the adoption of multidisciplinary care, improved staging techniques, standardised surgical procedures, and greater treatment personalisation [7]. Personalisation now extends beyond stage and conventional clinical risk to molecular characterisation of the tumour, including mismatch-repair and microsatellite instability status, which identifies the small subgroup in whom immune checkpoint inhibition can produce sustained responses, and to organ-preserving strategies in which sphincter preservation and quality of life are weighed alongside oncological outcome [3,7]. Despite these advances, population-level data documenting the real-world implementation of modern rectal cancer care strategies over extended periods remain scarce in the Baltic region. Few studies have described, within a single institutional dataset spanning two decades, how systemic changes—including MRI-based staging, multidisciplinary team (MDT) decision-making, total mesorectal excision (TME) standardisation, national colorectal cancer screening, and the introduction of total neoadjuvant treatment (TNT)—translated into measurable changes in stage at diagnosis, treatment patterns, and long-term survival. This evidence gap is particularly relevant for Lithuania and the wider Baltic context, where survival estimates have historically lagged behind Western European benchmarks [2,4,5].

The Hospital of the Lithuanian University of Health Sciences (LUHS) Kaunas Clinics is one of the largest rectal cancer referral centres in Lithuania, treating approximately one-third of all rectal cancer patients in the country. This study aimed to evaluate 20-year trends in clinical and pathological characteristics, treatment patterns, and survival outcomes, and to contextualise these findings within the broader trajectory of rectal cancer care implementation in Europe and North America.

## 2. Materials and Methods

This retrospective study was conducted at the Department of Oncology and Hematology, Hospital of the Lithuanian University of Health Sciences (LUHS) Kaunas Clinics (Kaunas, Lithuania). The study was approved by the Kaunas Regional Biomedical Research Ethics Committee (issue number BE-2-155, 31 August 2026).

### 2.1. Study Population

Eligible patients had histologically confirmed rectal cancer (International Classification of Diseases, 10th Revision, code C20), clinical stage I–III at diagnosis, and underwent surgical resection of the primary tumour between 2004 and 2023 at the Departments of Surgery. Patients with distant metastases (stage IV) at diagnosis and those with unknown vital status at the end of data collection were excluded. A total of 839 patients met the inclusion criteria. Because the study database was assembled from the institutional surgical registry, the cohort by design comprises only patients who ultimately underwent resection of the primary tumour; patients with stage I–III rectal cancer who progressed, died, or otherwise did not proceed to surgery after diagnosis were not recorded and could not be enumerated retrospectively. Dates of surgery were not captured in the database, so the interval between diagnosis and surgery could not be calculated. The consequences of this design for the comparison between cohorts, and the landmark sensitivity analysis performed to address them, are described in Section 2.5 and Section 4. Patient flow is summarised in Supplementary Figure S1.

### 2.2. Data Collection

Clinical and pathological data were retrospectively extracted from medical records in the LUHS Kaunas Clinics Patient Information System using a standardised data collection form. Recorded variables included sex, age, date of diagnosis (defined as the date of histological confirmation), date of death (obtained from the Lithuanian Cancer Registry), tumour characteristics, and treatment details. Perioperative treatment was classified as none, neoadjuvant (with or without subsequent adjuvant therapy), or adjuvant only. Radiotherapy was classified by modality as none, neoadjuvant radiotherapy, neoadjuvant chemoradiotherapy, adjuvant radiotherapy, or adjuvant chemoradiotherapy. Total neoadjuvant treatment (TNT) denotes the addition of preoperative systemic chemotherapy (induction FOLFOX) to the preoperative strategy and is therefore not a separate radiotherapy modality; all patients treated with TNT received radiotherapy with neoadjuvant intent and are counted both in the TNT row and within neoadjuvant chemoradiotherapy in Table 1. Variables with incomplete data are reported explicitly in Table 1 and in Section 3.

### 2.3. Cohort Definition

Patients were grouped into two cohorts based on year of diagnosis: 2004–2013 and 2014–2023. This division was chosen to reflect major changes in rectal cancer diagnostics and treatment protocols, and the nationwide implementation of the Lithuanian colorectal cancer screening programme starting in 2014.

### 2.4. Outcomes

Overall survival (OS) was defined as the time from diagnosis to death from any cause. Cancer-specific survival (CSS) was defined as the time from diagnosis to death attributed to cancer. Patients alive at the end of the study period were censored at the date of last follow-up (1 August 2024). Two pre-specified analyses were performed. In the primary analysis, follow-up was administratively truncated at 60 months for every patient so that the two cohorts were compared over an identical time horizon; patients still under observation at 60 months were censored at that point. In a secondary analysis, the complete available follow-up was used (up to 20 years in the 2004–2013 cohort and up to 10 years in the 2014–2023 cohort); this analysis is the sole source of the median survival times reported in Table 2, which cannot be estimated within a 60-month horizon. Five-year survival was estimated by the Kaplan–Meier method and therefore accounts for censoring: patients whose follow-up ended before 60 months were censored at their last contact and were not counted as five-year survivors.

### 2.5. Statistical Analysis

**Figure 1:**
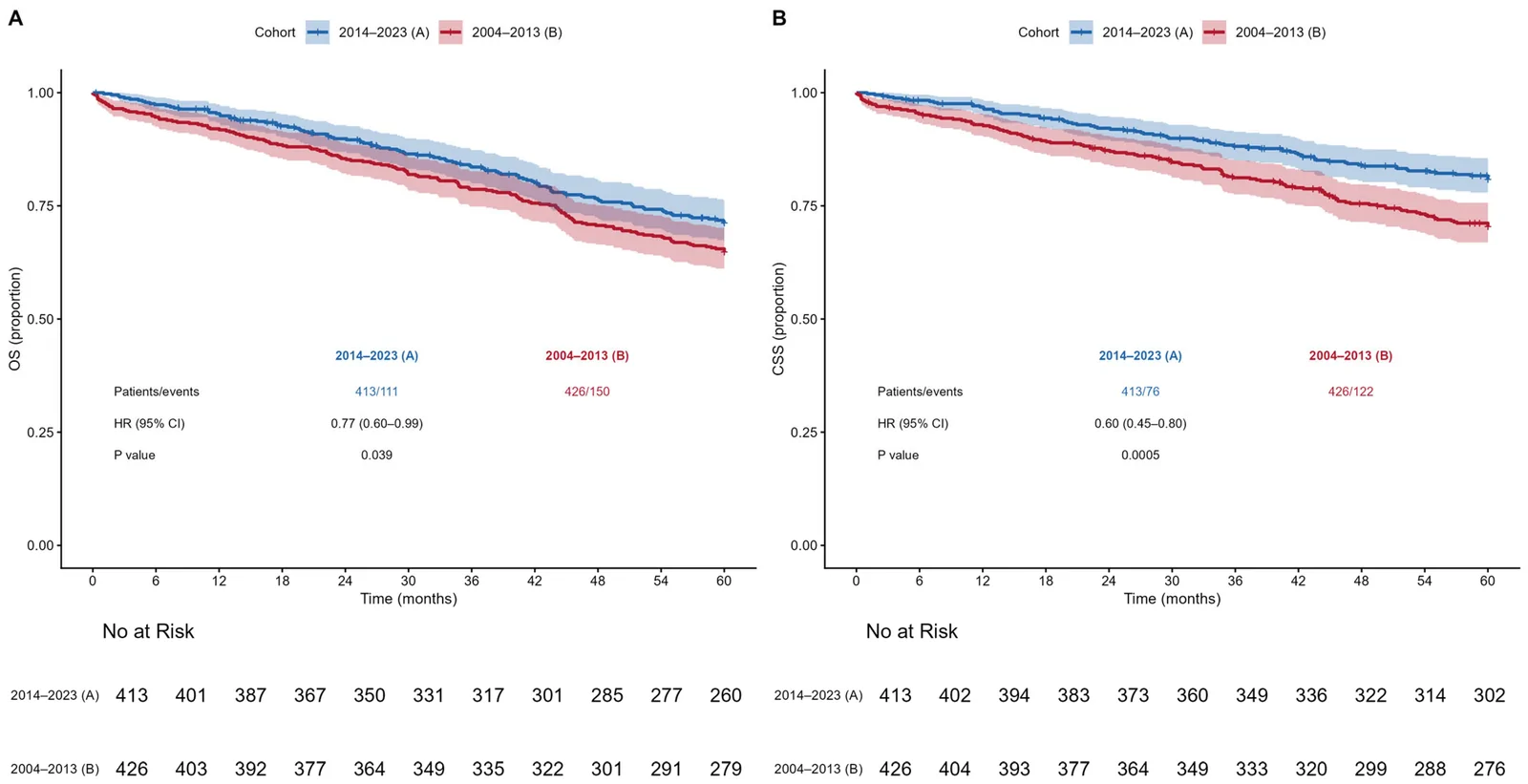
Kaplan–Meier curves for overall survival (**A**) and cancer-specific survival (**B**) in patients with stage I–III rectal cancer diagnosed in 2004–2013 and 2014–2023. Kaplan–Meier curves comparing survival between patients diagnosed in 2004–2013 (red line) and 2014–2023 (blue line). (**A**) Overall survival (OS): HR 0.77 (95% CI 0.60–0.99; *p* = 0.039). (**B**) Cancer-specific survival (CSS): HR 0.60 (95% CI 0.45–0.80; *p* = 0.0005). Number-at-risk tables are shown below each panel. Tick marks indicate censored observations. Follow-up is truncated at 60 months, the horizon of the primary analysis; the Kaplan–Meier estimates and numbers at risk shown account for censoring, and shaded bands denote 95% confidence intervals. Abbreviations: OS, overall survival; CSS, cancer-specific survival; HR, hazard ratio; CI, confidence interval.

Statistical analyses were performed using IBM SPSS Statistics for Windows, version 23.0 (IBM Corp., Armonk, NY, USA); Kaplan–Meier curves were generated using R version 4.6.0 (R Foundation for Statistical Computing, Vienna, Austria). Categorical variables were compared using the chi-square test or Fisher’s exact test when expected cell counts were small. The normality of continuous variables was assessed using the Kolmogorov–Smirnov test; non-normally distributed data were compared using the Mann–Whitney U test. Survival estimates, including 5-year survival rates and median survival times, were calculated using the Kaplan–Meier method, with between-group differences assessed by the log-rank test. Numbers at risk are reported at 6-month intervals beneath each panel of Figure 1. The crude event counts shown alongside the Kaplan–Meier estimates in Table 3 are descriptive only and were not used to estimate survival. Multivariable Cox proportional hazards regression models were used to identify independent predictors of OS and CSS and to estimate hazard ratios (HRs) with 95% confidence intervals (CIs), with a pre-specified set of covariates entered simultaneously, without stepwise or other data-driven variable selection: sex, age ≥ 65 years, cohort period, histological subtype (mucinous or signet-ring cell vs. adenocarcinoma), tumour grade (G1, G2, G3), tumour location (low, mid, or high rectum), clinical stage (III vs. I–II), and perioperative treatment (neoadjuvant, adjuvant only, or none). All models were fitted on complete cases. Missing values were confined to pathological stage, pT and pN (10 patients, 1.2%), and to radiotherapy modality (7 patients, 0.8%); neither variable entered the multivariable models, which were therefore estimated based on all 839 patients. Clinical rather than pathological stage was used for adjustment because pathological stage after neoadjuvant therapy is partly determined by treatment response and therefore acts as a mediator rather than a confounder of the cohort effect; adjusting for it would constitute over-adjustment. The proportional hazards assumption was assessed using scaled Schoenfeld residuals and was satisfied for both models (global *p* = 0.43 for OS and *p* = 0.45 for CSS; cohort term *p* = 0.75 and *p* = 0.97, respectively). A two-sided *p*-value < 0.05 was considered statistically significant. Exact two-sided *p*-values are reported throughout; values below 0.001 are shown as *p* < 0.001. To quantify the contribution of stage migration to observed survival differences, we conducted a sequential attenuation analysis in which clinical stage was added stepwise to the Cox model and the resulting change in the cohort period hazard ratio was expressed as a percentage of the crude log-hazard ratio. Formally, attenuation was calculated as (β_adjusted − β_crude)/β_crude × 100, where β_crude is the log-hazard ratio for cohort period in a model containing cohort period alone and β_adjusted the corresponding coefficient after clinical stage was added; the resulting values were 12.8% for CSS and 24.3% for OS. Clinical and pathological stage were recorded according to the TNM edition in force at the time of diagnosis (the 6th, 7th and 8th editions were used successively during the study period). Because the definitions of individual T and N categories changed between editions, analyses restricted to or adjusted for stage reduce, but do not fully eliminate, stage-migration bias. A sensitivity analysis restricted to patients with clinical stage III disease was performed to minimise the confounding effect of stage migration. To assess the potential influence of the interval between diagnosis and resection (immortal time), a landmark sensitivity analysis was additionally performed in which follow-up began 6 months after diagnosis and patients who died before that landmark were excluded. Pathological complete response (pCR), defined as pathological stage 0 (ypT0N0) after neoadjuvant treatment, was analysed as an additional binary endpoint using the chi-square test or Fisher’s exact test, as appropriate. The study was reported in accordance with the STROBE guidelines for observational studies.

## 3. Results

### 3.1. Patient Population and Follow-Up

Of 917 patients with rectal cancer who underwent primary tumour resection at LUHS Kaunas Clinics between 2004 and 2023, 78 were excluded due to stage IV disease and/or unknown vital status, yielding a final cohort of 839 patients: 426 in the 2004–2013 cohort and 413 in the 2014–2023 cohort. The median follow-up was 142 months (IQR 80–190) and 58 months (IQR 30–82), respectively. During the observation period, 323 deaths (75.8%) occurred in the 2004–2013 cohort and 163 (39.5%) in the 2014–2023 cohort; these crude rates reflect the substantially longer follow-up in the earlier cohort. Within the pre-specified 60-month horizon of the primary analysis there were 150 deaths (35.2%) in the 2004–2013 cohort and 111 (26.9%) in the 2014–2023 cohort, of which 122 and 76, respectively, were attributed to rectal cancer. Deaths from causes other than rectal cancer numbered 31 (7.3%) and 37 (9.0%), so that the share of all deaths not attributable to rectal cancer rose from 20.7% in 2004–2013 to 33.3% in 2014–2023. No patient in the 2004–2013 cohort had less than 60 months of follow-up for overall survival, whereas 42 patients (10.2%) in the 2014–2023 cohort were censored before 60 months. Patient flow, including reasons for exclusion, is shown in Supplementary Figure S1. Data were complete for every variable entered into the survival models; pathological stage, pT and pN were unavailable for 10 patients (1 in 2004–2013 and 9 in 2014–2023) and radiotherapy modality was not recorded for 7 patients (all in 2014–2023).

### 3.2. Baseline Characteristics

Demographic characteristics were comparable between cohorts, with a slightly higher proportion of men in the 2004–2013 cohort (61.7% vs. 56.7%, *p* = 0.134). The median age at diagnosis was 68 years. Most tumours were adenocarcinomas (93.2%), located in the lower (46.9%) or middle (37.5%) rectum, and classified as grade 2 (66.8%) (Table 1).

### 3.3. Clinical and Pathological Staging

A significant shift toward earlier-stage disease was observed in the 2014–2023 cohort (Table 1). Clinical stage I diagnoses increased from 2.4% to 12.6%, and the proportion of tumours confined to the rectal wall (cT1–T2) rose from 6.3% to 16.7%. The rate of clinically node-negative disease (cN0) was higher in the later cohort (27.6% vs. 20.0%; *p* = 0.01). Pathological staging demonstrated concordant trends. The proportion of stage 0–I disease increased from 21.8% to 33.9%, pT0–T2 tumours rose from 27.0% to 45.7%, and pN0 status increased from 59.4% to 70.0%, while pN2 disease decreased from 12.2% to 5.3%.

### 3.4. Treatment Patterns

A substantial shift toward intensified preoperative strategies occurred between the two eras (Table 1). Neoadjuvant or perioperative treatment increased from 37.1% to 55.2% (*p* < 0.001), while surgery alone decreased from 42.7% to 30.3% (*p* < 0.001). This transition was accompanied by a more than threefold increase in neoadjuvant chemoradiotherapy (74/426 (17.4%) to 224/413 (54.2%); *p* < 0.001) and by the introduction of total neoadjuvant treatment (TNT), administered to 51/413 patients (12.3%) in the later cohort (*p* < 0.001). Conversely, standalone neoadjuvant radiotherapy declined from 84/426 (19.7%) to 8/413 (1.9%), and adjuvant radiotherapy from 24/426 (5.6%) to 3/413 (0.7%) (both *p* < 0.001). The proportion of patients receiving no radiotherapy decreased from 201/426 (47.2%) to 134/413 (32.4%) (*p* < 0.001), while adjuvant chemoradiotherapy use remained stable (43/426, 10.1% vs. 37/413, 9.0%); radiotherapy modality was not recorded for 7/413 patients (1.7%) in the later cohort. TNT is not a separate radiotherapy modality but the addition of preoperative systemic chemotherapy to the preoperative strategy; all 51 patients who received TNT were treated with radiotherapy of neoadjuvant intent and are therefore counted within neoadjuvant chemoradiotherapy above. Neoadjuvant regimens included long-course chemoradiotherapy, short-course radiotherapy (5 × 5 Gy), and induction FOLFOX; adjuvant treatment shifted from the Mayo regimen to FOLFOX.

### 3.5. Stage-Specific Survival Outcomes

Table 3 presents the 5-year survival outcomes stratified by stage. For stage III disease, neoadjuvant and/or adjuvant treatment (NAT/AT) utilisation increased from 204/353 (57.8%) to 251/299 (83.9%) (*p* < 0.001), accompanied by a significant improvement in 5-year CSS (67.9% to 77.3%; log-rank *p* = 0.009), while the improvement in 5-year OS did not reach significance (62.3% to 67.6%; log-rank *p* = 0.15). For stage II disease, NAT/AT rates decreased from 40/63 (63.5%) to 29/62 (46.8%) (*p* = 0.060), reflecting more selective treatment; despite this de-escalation, 5-year OS and CSS showed non-significant upward trends (OS: 74.6% to 78.1%, log-rank *p* = 0.59; CSS: 79.1% to 88.3%, log-rank *p* = 0.16). Stage I patients maintained excellent outcomes in both cohorts, with CSS consistently above 90%.

### 3.6. Overall Survival Outcomes

Patients in the 2014–2023 cohort demonstrated significantly improved survival (Table 2). Five-year OS increased from 64.8% (95% CI 60.0–69.1%) to 71.3% (95% CI 66.4–75.5%) (log-rank *p* = 0.039; univariate HR 0.77; 95% CI 0.60–0.99; *p* = 0.039). Median OS was 95.0 months (95% CI 80.2–109.8) in the earlier cohort and was not reached in the later cohort. Five-year CSS improved from 70.4% (95% CI 65.7–74.6%) to 80.8% (95% CI 76.6–84.4%) (log-rank *p* = 0.0005; univariate HR 0.60; 95% CI 0.45–0.80; *p* = 0.0005), with a median CSS of 201.0 months (95% CI 150.9–251.1) versus not reached. Kaplan–Meier curves demonstrated sustained separation favouring the 2014–2023 cohort for both OS and CSS (Figure 1A,B). In a sensitivity analysis restricted to clinical stage III patients (n = 353 vs. 299), five-year CSS remained significantly higher in the 2014–2023 cohort (77.3% vs. 67.9%; log-rank *p* = 0.009), confirming that the CSS benefit persists even when stage migration is minimised. In the landmark sensitivity analysis beginning 6 months after diagnosis (35 patients excluded because of death before the landmark, 23 in the earlier and 11 in the later cohort, plus one patient with censoring before the landmark), the cancer-specific survival advantage of the 2014–2023 cohort was essentially unchanged (HR 0.65; 95% CI 0.48–0.88; log-rank *p* = 0.006; HR 0.64; 95% CI 0.45–0.89; *p* = 0.008 after multivariable adjustment), whereas the difference in overall survival remained non-significant (HR 0.83; 95% CI 0.64–1.07; *p* = 0.15), indicating that immortal time between diagnosis and surgery does not account for the observed cancer-specific difference.

### 3.7. Survival by Pathological Stage

Survival was additionally examined within pathological stage strata, which are reported in the Discussion. Five-year cancer-specific survival improved from 81.1% to 92.8% in pathological stage I (log-rank *p* = 0.018) and from 73.8% to 83.8% in pathological stage II (*p* = 0.026). Differences in pathological stage 0 (90.9% vs. 92.3%; *p* = 0.92) and pathological stage III (61.0% vs. 64.1%; *p* = 0.56) were not statistically significant. Corresponding five-year overall survival estimates were 90.9% vs. 91.7% (*p* = 0.97) for stage 0, 72.0% vs. 80.9% (*p* = 0.15) for stage I, 69.8% vs. 74.5% (*p* = 0.32) for stage II and 55.5% vs. 55.0% (*p* = 0.98) for stage III. Because pathological stage after neoadjuvant therapy reflects treatment response, and because the TNM edition in use changed during the study period, these strata are not fully comparable between eras and the estimates are presented as descriptive.

### 3.8. Multivariable Analysis

Table 4 presents multivariable Cox proportional hazards models for OS and CSS. In univariate analysis, the 2014–2023 cohort was associated with a 23% lower OS mortality risk (HR 0.77; 95% CI 0.60–0.99; *p* = 0.039) and a 40% lower CSS mortality risk (HR 0.60; 95% CI 0.45–0.80; *p* = 0.0005). In the multivariable OS model, the cohort period effect was attenuated to non-significance after multivariable adjustment (HR 0.84; 95% CI 0.64–1.10; *p* = 0.20); sequential attenuation analysis showed that clinical stage explained approximately 24% of the crude cohort log-hazard ratio for OS. In contrast, in the multivariable CSS model, the cohort period effect remained statistically significant after full adjustment (HR 0.618; 95% CI 0.450–0.849; *p* = 0.003), indicating that the survival benefit in cancer-specific mortality is not explained by stage migration alone. Age ≥ 65 years (OS: HR 2.25, 95% CI 1.67–3.03; CSS: HR 2.03, 95% CI 1.46–2.83) and clinical stage III (OS: HR 1.93, 95% CI 1.35–2.76; CSS: HR 2.04, 95% CI 1.31–3.16) were independent predictors of increased mortality in both models. In the multivariable OS model, high-grade tumours (grade 3) were additionally associated with worse OS (HR 1.90; 95% CI 1.22–2.97; *p* = 0.005). Sex, tumour morphology, tumour localisation, and perioperative treatment type were not significantly associated with OS in multivariable analysis. Among patients treated with neoadjuvant chemoradiotherapy or TNT, pCR (ypT0N0) was observed in 24/224 (10.7%) patients in 2014–2023 and 8/74 (10.8%) in 2004–2013 (*p* = 0.98); across all neoadjuvantly treated patients, the rates were 25/228 (11.0%) and 11/158 (7.0%), respectively (*p* = 0.18). These denominators differ from the pathological stage 0 counts in Table 1 (26/413 and 11/426), which include all patients with pathological stage 0 irrespective of the treatment received. Other measures of response to preoperative treatment were similarly stable. Among neoadjuvantly treated patients, ypT0–T2 disease was recorded in 57/157 (36.3%) in 2004–2013 and 91/219 (41.6%) in 2014–2023 (*p* = 0.45), ypN0 in 108/157 (68.8%) and 150/219 (68.5%) (*p* = 0.60), and pathological stage 0–I in 49/158 (31.0%) and 61/228 (26.8%) (*p* = 0.36).

## 4. Discussion

This study reports 20-year trends in real-world rectal cancer management at the tertiary centre treating approximately one-third of all rectal cancer patients in Lithuania. Progressive implementation of MRI-based staging, multidisciplinary team (MDT) decision-making, total mesorectal excision (TME) standardisation, national colorectal cancer screening, and total neoadjuvant treatment (TNT) was associated with a shift toward earlier-stage diagnosis, intensified preoperative strategies, and improved long-term survival. Five-year overall survival increased from 64.8% to 71.3%, and cancer-specific survival improved from 70.4% to 80.8%—trends consistent with Western European registries but documented here from a Baltic perspective for the first time in over two decades.

### 4.1. Contextualisation Against International Benchmarks

These gains are notable when viewed against established benchmarks. The CONCORD-3 study reported 5-year net survival of 50–59% for Lithuania and other Baltic and Central European countries during 2010–2014, compared with 60–69% in most Western European countries [5]. However, CONCORD-3 did not analyse rectal cancer separately; the reported survival figures were based on a combined ICD-10 C20–C21 group, which included patients with rectal and anal cancers. Stage-specific data are consistent with a strong gradient in survival, ranging from approximately 90% for stage I to just over 10% for stage IV disease [6]. In the United States, the Surveillance, Epidemiology, and End Results (SEER) database reports an overall 5-year survival of 67%, with stage-dependent variation from 90% for localised disease to 17% for metastatic disease [8]. In contrast, an analysis of the National Cancer Database demonstrated that the median overall survival for the overall patient cohort increased from 83.1 months (2004–2007) to 92.1 months (2012–2015), likely reflecting advances in staging, multimodal treatment, and surgical technique [9]. The present findings suggest that Lithuania has followed a comparable implementation trajectory, progressing from a lower baseline consistent with CONCORD-3 estimates for the Baltic region and achieving outcomes in 2014–2023 that appear to be approaching those of Western European centres during comparable periods.

### 4.2. Diagnostic and Screening Advances

The increased proportion of early-stage diagnoses in the 2014–2023 cohort likely reflects improvements in diagnostic imaging and the national screening programme. Endorectal ultrasound was introduced at our institution in 2002, followed by pelvic MRI as a standard staging modality in 2008. Since approximately 2016, computed tomography of the chest, abdomen, and pelvis has been routinely performed for distant metastasis detection. A pilot colorectal cancer screening programme [10], initiated in 2009 and expanded nationally by 2014, may have contributed to earlier detection.

### 4.3. Evolution of Treatment Strategy

Rectal cancer management has evolved from a surgery-first approach to a multidisciplinary strategy integrating neoadjuvant and adjuvant therapies, advanced imaging, and individualised treatment planning, including organ-preserving and watch-and-wait approaches for selected patients [7]. At our institution, this evolution followed a defined trajectory. Before 2006, treatment typically began with surgery followed by adjuvant radiotherapy or chemoradiation. The introduction of neoadjuvant chemoradiotherapy and TME standardisation in 2006 marked a pivotal shift, informed by the CAO/ARO/AIO-94 trial demonstrating reduced local recurrence (6% vs. 13%) and lower toxicity with preoperative chemoradiotherapy [11]. Routine MDT discussions were formalised from 2013, enabling standardised MRI-based staging and optimised treatment selection. Adjuvant protocols were further refined following the ADORE trial, which showed that oxaliplatin-based chemotherapy (FOLFOX) significantly improved disease-free survival in patients with persistent pathological stage II–III disease after neoadjuvant chemoradiotherapy [12]. In 2018, the Lithuanian Ministry of Health designated high-volume colorectal cancer centres with a minimum threshold of 50 rectal cancer surgeries annually, consolidating surgical expertise at centres including our institution [13]. The chronology of these milestones is summarised in Supplementary Materials, Table S1; the developments listed there are presented as possible contributors to the observed trends and were not evaluated individually in this study.

These developments were accompanied by a significant increase in pre- and perioperative therapy use (55.2% vs. 37.1%; *p* < 0.001). Most strikingly, the proportion of clinical stage III patients receiving neoadjuvant chemoradiotherapy or TNT rose from 15.9% to 70.9% (*p* < 0.001). In the multivariable OS model, the cohort period effect was attenuated to non-significance after adjustment for clinical stage and other covariates (HR 0.84; 95% CI 0.64–1.10; *p* = 0.20). Sequential attenuation analysis demonstrated that clinical stage explained approximately 13% of the crude cohort log-hazard ratio for CSS, indicating that stage migration accounts for only a minor fraction of the observed cancer-specific survival improvement. In contrast, the cohort period effect remained independently significant in the multivariable CSS model (HR 0.618; 95% CI 0.450–0.849; *p* = 0.003), consistent with a real improvement in cancer-specific outcomes, although residual confounding from unmeasured prognostic factors (see Strengths and Limitations) cannot be excluded. The attenuation of the cohort effect in OS—but not CSS—is biologically interpretable: OS in an elderly population is substantially influenced by competing causes of death unrelated to cancer, which dilute the cohort signal after multivariable adjustment. This is directly visible in the cause-specific mortality within 60 months: deaths not attributable to rectal cancer accounted for 20.7% of all deaths in the earlier cohort and 33.3% in the later one, so that a larger share of the mortality observed in 2014–2023 lies outside the reach of oncological treatment. The observed survival gains should therefore be interpreted as the cumulative result of concurrent system-level changes rather than attributable to any single intervention, consistent with the CONCORD-3 analysis attributing survival improvements in countries with historically lower benchmarks to the collective effect of diagnostic modernisation, treatment standardisation, and multidisciplinary care organisation [5]. In particular, the stability of pathological complete response rates shows only that per-patient tumour responsiveness did not change between eras; it does not identify which of the concurrent changes in screening, imaging, surgery, multidisciplinary organisation and systemic therapy contributed to the survival gain, and no causal attribution to any of them is possible from these data.

Comparable patterns have been documented internationally. The Dutch TME programme demonstrated that surgical standardisation combined with neoadjuvant radiotherapy substantially reduced local recurrence nationwide [7]. In the US, increasing use of chemotherapy, sphincter-saving surgery, minimally invasive techniques, and neoadjuvant radiotherapy between 2004 and 2016 closely mirrors the shift observed in this cohort [9]. Randomised evidence has since established the role of intensified preoperative strategies: the RAPIDO trial demonstrated reduced disease-related treatment failure with short-course radiotherapy followed by chemotherapy [14], and PRODIGE 23 showed improved disease-free survival with induction chemotherapy before chemoradiotherapy [15]; these trials underpin the TNT protocols adopted at our institution. An Italian single-centre study similarly reported improving 5-year overall survival from 1980 to 2010, particularly in patients with advanced-stage tumours [16].

### 4.4. Stage-Specific and Subgroup Observations

Notably, stage II patients demonstrated improved survival despite reduced neoadjuvant therapy administration (63.5% to 46.8%), suggesting that enhanced diagnostic precision and more accurate clinical staging enabled better patient selection and appropriately tailored treatment intensity without compromising outcomes. To assess whether the overall survival improvement could be explained by stage migration (the Will Rogers phenomenon), we examined survival within each clinical and pathological stage stratum. Among clinical stage III patients—the largest and most homogeneous group—five-year CSS increased from 67.9% to 77.3% (log-rank *p* = 0.009; HR 0.66, 95% CI 0.49–0.90), and the cohort period remained an independent predictor in multivariable CSS analysis restricted to this group. Together with the pathological stage-stratified estimates reported in Section 3, these within-stage improvements indicate that the survival gains are not solely a reclassification artefact, although changes in TNM edition mean that stage strata are not perfectly comparable across the two eras. The adoption of TNT increased from 0% to 12.3% between cohorts, aligning with evolving ESMO guidelines advocating TNT for high-risk locally advanced rectal cancer [3]. By delivering systemic chemotherapy alongside radiotherapy before surgery, TNT aims to enhance pathological complete response rates and reduce distant metastasis risk. In this cohort, pCR (ypT0N0) among patients receiving neoadjuvant chemoradiotherapy or TNT was 10.7% in 2014–2023 versus 10.8% in 2004–2013 (*p* = 0.98). The apparent rise when expressed as a proportion of the whole cohort (2.6% to 6.3%) is entirely attributable to the far larger share of patients receiving neoadjuvant therapy, not to improved tumour responsiveness. Its implementation at our institution reflects the successful integration of evidence-based multimodal strategies into routine practice. Other measures of response to preoperative treatment, reported in Section 3, were likewise stable between eras, indicating that the survival gain did not follow from a change in per-patient tumour behaviour. Because screening, MRI-based staging, surgical technique, multidisciplinary organisation, systemic therapy and supportive care all evolved concurrently, the present data cannot isolate wider neoadjuvant delivery as the principal driver; the gain is more appropriately interpreted as the combined effect of these parallel improvements in the care pathway. Practice has since moved further towards individualisation: molecular and genetic characterisation of the tumour, in particular mismatch-repair and microsatellite instability status, now guides selection for immune checkpoint inhibition, and functional endpoints—sphincter preservation, low anterior resection syndrome and health-related quality of life—are increasingly reported alongside survival [3,7]. Neither molecular data nor functional outcomes were recorded systematically over the present study period; their evaluation in the 2014–2023 cohort, together with a detailed analysis of surgical technique and of response to neoadjuvant treatment, is a priority for future work at our centre. Age ≥ 65 years, stage III disease, and high-grade (G3) tumours remained independent prognostic factors for poorer survival, consistent with prior studies [17,18,19,20,21] and underscoring the need for individualised treatment and closer surveillance in these high-risk subgroups.

### 4.5. Strengths and Limitations

This study is based on a large, well-defined cohort with histologically confirmed rectal cancer treated at a single centre over 20 years, with verified mortality data from the Lithuanian Cancer Registry. Detailed clinical, pathological, and treatment data enabled subgroup analyses and multivariable adjustment for key prognostic factors. Limitations include the retrospective design with inherent risk of data inaccuracy or incompleteness. Although conducted at a single centre, this institution treats approximately one-third of national rectal cancer cases, partially mitigating generalisability concerns. The historical-cohort design introduces temporal confounding, as multiple factors changed simultaneously between eras. Shorter follow-up in the 2014–2023 cohort (up to 10 vs. 20 years) may underestimate the true survival benefit. Importantly, while stage migration may contribute to apparent improvements in stage-specific survival, our sequential attenuation analysis demonstrated that clinical stage accounts for only approximately 13% of the crude CSS cohort hazard ratio, and the survival benefit in cancer-specific mortality remained significant in multivariable analysis (HR 0.618; *p* = 0.003). The portion of the cohort effect not explained by clinical stage should be interpreted as reflecting a combination of real improvement in care and any residual confounding by unmeasured factors, rather than improvement alone. Furthermore, the CSS improvement was confirmed in sensitivity analyses restricted to clinical stage III patients, reducing the likelihood that the findings represent a Will Rogers artefact. Data on local recurrence, circumferential resection margin positivity, and permanent stoma rates were unavailable for the full study period. Functional and patient-reported outcomes—sphincter preservation, low anterior resection syndrome and health-related quality of life—were likewise not recorded, so this analysis cannot address the trade-off between survival and function that is central to contemporary rectal cancer care. A further limitation concerns cohort selection. Eligibility required resection of the primary tumour while survival was measured from diagnosis, so every included patient necessarily survived the interval between diagnosis and surgery. Because that interval lengthened as neoadjuvant therapy became more widely used, this immortal time may favour the later cohort, and patients who progressed or died before reaching surgery are absent from both cohorts and could not be identified retrospectively. The landmark analysis starting 6 months after diagnosis, in which the cancer-specific survival advantage was essentially unchanged (HR 0.65; 95% CI 0.48–0.88), suggests that this bias does not explain the observed difference, but it cannot be excluded entirely. Staging was also performed according to the TNM edition in force at the time of diagnosis, and category definitions changed between the 6th, 7th and 8th editions; stage-stratified and stage-adjusted analyses therefore mitigate but do not fully remove stage-migration bias. In addition, the marked shift in recorded tumour location (low rectal tumours: 34.7% to 59.3%) and in tumour grade (grade 1: 38.7% to 11.6%) most likely reflects changes in radiological and pathological reporting conventions rather than true biological change. The apparent increase in low rectal tumours coincided with routine MRI-based measurement of tumour distance from the anal verge from 2008 onwards, which reclassified many tumours previously recorded as mid rectal to low rectal, and with the centralisation of low rectal cancer surgery at our institution. The decline in grade 1 tumours parallels the WHO move to two-tier grading, under which most formerly grade 1 tumours are now reported as low-grade or grade 2, consistent with the concurrent rise in grade 2 tumours from 55.2% to 78.7%. These variables should therefore be interpreted with caution. Finally, median follow-up in the later cohort was 58 months, so all survival comparisons were truncated at 60 months; longer-term outcomes for the 2014–2023 cohort remain to be confirmed. Several potentially important prognostic variables were unavailable for the full study period and could not be included in the multivariable models, including comorbidity burden and ASA physical status, postoperative complications, lymphovascular and perineural invasion, molecular and biomarker data, completion of adjuvant chemotherapy, and disease recurrence. Changes in diagnostic practice and disease coding, the evolution of pathological reporting standards, and advances in perioperative and supportive care over the study period may likewise have contributed to the observed differences. Despite these limitations, this represents the longest institutional implementation dataset from Lithuania reported to date.

## 5. Conclusions

Over 20 years, progressive adoption of modern rectal cancer management strategies at a major Lithuanian tertiary centre was associated with significantly improved cancer-specific survival (5-year CSS: 70.4% to 80.8%; univariate HR 0.60; 95% CI 0.45–0.80; *p* = 0.0005; multivariable HR 0.618; 95% CI 0.450–0.849; *p* = 0.003) and overall survival (5-year OS: 64.8% to 71.3%; univariate HR 0.77; 95% CI 0.60–0.99; log-rank *p* = 0.039). Sequential attenuation analysis confirmed that stage migration explains only a minor fraction (~13%) of the observed CSS benefit, while sensitivity analyses in clinical stage III patients corroborated the genuine nature of survival improvements (CSS: 67.9% to 77.3%; *p* = 0.009). Pathological complete response rates among neoadjuvantly treated patients remained stable at approximately 11% (10.8% vs. 10.7%; *p* = 0.98), indicating that the survival gain did not arise from greater per-patient tumour responsiveness. Given the many concurrent changes over the study period—earlier-stage diagnosis, MRI-based staging, refinements in surgical technique, formalised multidisciplinary care, wider and better-targeted neoadjuvant and systemic therapy, and improved supportive care—the improvement is best understood as the cumulative result of these system-level developments rather than being attributable to any single intervention. These improvements coincided with a shift towards earlier-stage diagnosis, broader adoption of neoadjuvant treatment strategies, the introduction of TNT, formalisation of MDT care, and the implementation of a nationwide screening programme. These findings provide real-world evidence from the Baltic region that systematic adoption of modern rectal cancer management pathways is associated with meaningful, measurable improvements in patient outcomes. As an observational study comparing two historical cohorts, this analysis cannot establish causation, and residual confounding cannot be excluded; nonetheless, the consistency of the association across overall and cancer-specific survival, within-stage strata, and multivariable models supports its robustness.

## Figures and Tables

**Table 1:** Demographic and clinical characteristics at diagnosis.

Characteristic	2004–2013 Cohort	2014–2023 Cohort	Total	*p*-Value
	*n* = 426	*n* = 413	*n* = 839	
Men, *n* (%)	263 (61.74)	234 (56.66)	497 (59.24)	0.134
Age at diagnosis, years				0.612
median	68.0	69.0	68.0	
mean (SD)	66.99 (9.69)	67.35 (10.12)	67.17 (9.90)	
range	30–86	32–88	30–88	
Tumor characteristics				
Tumor morphology (histology), *n* (%)				0.265
Adenocarcinoma	393 (92.25)	389 (94.19)	782 (93.21)	
Mucinous/Signet ring cell carcinoma	33 (7.75)	24 (5.81)	57 (6.79)	
Tumor location, *n* (%)				<0.001
Low rectal (0–5 cm)	148 (34.75)	245 (59.32)	393 (46.85)	
Mid rectal (6–10 cm)	216 (50.70)	99 (23.97)	315 (37.54)	
High rectal (11–15 cm)	62 (14.55)	69 (16.71)	131 (15.61)	
Tumor grade, *n* (%)				<0.001
Grade 1	165 (38.73)	48 (11.62)	213 (25.39)	
Grade 2	235 (55.16)	325 (78.69)	560 (66.75)	
Grade 3	26 (6.10)	40 (9.69)	66 (7.86)	
Clinical staging (cTNM)				
Clinical stage (overall), *n* (%)				<0.001
I	10 (2.35)	52 (12.59)	62 (7.39)	
II	63 (14.79)	62 (15.01)	125 (14.90)	
III	353 (82.86)	299 (72.40)	652 (77.71)	
cT stage, *n* (%)				<0.001
cT1	0 (0)	14 (3.4)	14 (1.7)	
cT2	27 (6.3)	55 (13.3)	82 (9.8)	
cT3	316 (74.2)	271 (65.6)	587 (70.0)	
cT4	83 (19.5)	73 (17.7)	156 (18.5)	
cN stage, *n* (%)				<0.001
cN0	85 (20.0)	114 (27.6)	199 (23.7)	
cN1	238 (55.8)	129 (31.2)	367 (43.7)	
cN2	103 (24.2)	168 (40.7)	271 (32.3)	
cNx	0	2 (0.5)	2 (0.3)	
Pathological staging (pTNM)				
Pathological stage (overall), *n* (%)				<0.001
0	11 (2.6)	26 (6.3)	37 (4.4)	
I	82 (19.2)	114 (27.6)	196 (23.4)	
II	159 (37.3)	149 (36.1)	308 (36.7)	
III	173 (40.6)	115 (27.8)	288 (34.3)	
Unknown	1 (0.3)	9 (2.2)	10 (1.2)	
pT stage, *n* (%)				<0.001
pT0	11 (2.6)	33 (8.0)	44 (5.2)	
pT1	15 (3.5)	51 (12.3)	66 (7.9)	
pT2	89 (20.9)	105 (25.4)	194 (23.1)	
pT3	276 (64.8)	203 (49.2)	479 (57.1)	
pT4	34 (8.0)	12 (2.9)	46 (5.5)	
pTx	1 (0.2)	9 (2.2)	10 (1.2)	
pN stage, *n* (%)				<0.001
pN0	253 (59.4)	289 (70.0)	542 (64.6)	
pN1	120 (28.2)	93 (22.5)	213 (25.4)	
pN2	52 (12.2)	22 (5.3)	74 (8.8)	
pNx	1 (0.2)	9 (2.2)	10 (1.2)	
Neoadjuvant/adjuvant treatment, *n* (%)				<0.001
No peri-op adjuvant treatment,	182 (42.7)	125 (30.3)	307 (36.6)	
Neoadjuvant or neoadjuvant + adjuvant treatment,	158 (37.1)	228 (55.2)	386 (46.0)	
Adjuvant treatment,	86 (20.2)	60 (14.5)	146 (17.4)	
Total neoadjuvant treatment, *n* (%)	0 (0)	51 (12.34)	51 (6.1)	<0.001
Radiotherapy, *n* (%)				<0.001
No radiotherapy	201 (47.18)	134 (32.45)	335 (39.93)	
Neoadjuvant radiotherapy	84 (19.71)	8 (1.94)	92 (10.96)	
Neoadjuvant chemoradiotherapy	74 (17.37)	224 (54.24)	298 (35.52)	
Adjuvant radiotherapy	24 (5.63)	3 (0.73)	27 (3.22)	
Adjuvant chemoradiotherapy	43 (10.09)	37 (8.96)	80 (9.54)	
Unknown	0 (0)	7 (1.69)	7 (0.83)	

Data are *n* (%) unless otherwise stated. *p*-values refer to the overall comparison between the two cohorts for each variable and were obtained with Pearson’s chi-square test (Fisher’s exact test for total neoadjuvant treatment) for categorical variables and the Mann–Whitney U test for age; exact values are given, and values below 0.001 are shown as *p* < 0.001. Total neoadjuvant treatment denotes the addition of preoperative systemic chemotherapy (induction FOLFOX) to the preoperative strategy and is not a separate radiotherapy modality; patients receiving it are also counted in the radiotherapy categories according to the modality recorded for them. Percentages are column percentages within each cohort.

**Table 2:** Survival outcomes for stage I–III rectal cancer patients across two treatment eras (2004–2013 vs. 2014–2023): five-year estimates from the 60-month primary analysis and median survival from the complete follow-up analysis.

Survival Outcome	2004–2013 Cohort	2014–2023 Cohort	HR (95% CI) *	*p*-Value *
Overall Survival (OS)				
5-year rate, % (95% CI)	64.8 (60.0–69.1)	71.3 (66.4–75.5)	0.77 (0.60–0.99)	0.039
Median survival, months (95% CI)	95.0 (80.2–109.8)	NR (NR–NR)	-	0.039
Cancer-Specific Survival (CSS)				
5-year rate, % (95% CI)	70.4 (65.7–74.6)	80.8 (76.6–84.4)	0.60 (0.45–0.80)	0.0005
Median survival, months (95% CI)	*201.0* (150.9–251.1)	NR (NR–NR)	-	0.0005

**Abbreviations:** CI, confidence interval; HR, hazard ratio; NR, not reached; * Hazard ratios (HRs) and *p*-values for 5-year rates were derived from the Cox proportional hazards model (reference: 2004–2013 cohort). *p*-values for median survival were determined by the log-rank test. Five-year rates, hazard ratios and their *p*-values were derived from the pre-specified primary analysis, in which follow-up was administratively truncated at 60 months for all patients; they are Kaplan–Meier point estimates at 60 months and account for censoring. Median survival could not be estimated within that horizon and is therefore taken from the secondary analysis using the complete available follow-up (up to 20 years in the 2004–2013 cohort and up to 10 years in the 2014–2023 cohort). The two sets of estimates are derived from different follow-up horizons and are not directly comparable.

**Table 3:** Five-year (60-month) survival outcomes by clinical stage and diagnostic period (2004–2013 vs. 2014–2023).

	2004–2013 Cohort					2014–2023 Cohort				
	Cancer-Specific Survival		Overall Survival			Cancer-Specific Survival		Overall Survival		
	Alive or Censored *n* = 304	Died ≤ 60 mo *n* = 122	Alive or Censored *n* = 276	Died ≤ 60 mo *n* = 150	Total *n* = 426	Alive or Censored *n* = 337	Died ≤ 60 mo *n* = 76	Alive or Censored *n* = 302	Died ≤ 60 mo *n* = 111	Total *n* = 413
Cancer stage	***n*, (%)**	***n*, (%)**	***n*, (%)**	***n*, (%)**	***n*, (%)**	***n*, (%)**	***n*, (%)**	***n*, (%)**	***n*, (%)**	***n*, (%)**
Stage I	10 (100.0)	0 (0.0)	9 (90.0)	1 (10.0)	10 (2.4)	48 (92.3)	4 (7.7)	43 (82.7)	9 (17.3)	52 (12.6)
5-year KM	OS 90.0 (47.3–98.5); CSS 100.0 (no cancer-related death)					OS 82.6 (69.3–90.6); CSS 92.2 (80.5–97.0)				
(N)AT	0	0	0	0	0	6/52 (11.5)	2/52 (3.9)	6/52 (11.5)	2/52 (3.9)	8/52 (15.4)
Stage II	50 (79.4)	13 (20.6)	47 (74.6)	16 (25.4)	63 (14.7)	55 (88.7)	7 (11.3)	49 (79.0)	13 (21.0)	62 (15.0)
5-year KM	OS 74.6 (61.9–83.6); CSS 79.1 (66.6–87.3)					OS 78.1 (65.3–86.7); CSS 88.3 (77.0–94.3)				
(N)AT	31/63 (49.2)	9/63 (14.3)	30/63 (47.6)	10/63 (15.9)	40/63 (63.5)	25/62 (40.3)	4/62 (6.5)	23/62 (37.1)	6/62 (9.7)	29/62 (46.8)
Stage III	244 (69.1)	109 (30.9) *	220 (62.3)	133 (37.7) **	353 (82.9)	234 (78.3)	65 (21.7) *	210 (70.2)	89 (29.8) **	299 (72.4)
5-year KM	OS 62.3 (57.0–67.1); CSS 67.9 (62.7–72.6)					OS 67.6 (61.7–72.8); CSS 77.3 (72.0–81.7)				
(N)AT	144/353 (40.8)	60/353 (17.0)	136/353 (38.5)	68/353 (19.3)	204/353 (57.8)	196/299 (65.5)	55/299 (18.4)	181/299 (60.5)	70/299 (23.4)	251/299 (83.9)

Counts are crude numbers of patients within each cohort and stage during the first 60 months after diagnosis. The “alive or censored” columns include patients whose follow-up ended before 60 months and who are therefore not known to be alive at that time point; five-year survival should be read from the rows labelled “5-year KM”, which give the Kaplan–Meier estimate of five-year survival as a percentage with its 95% confidence interval and account for censoring. * *p* = 0.009 (cancer-specific survival) and ** *p* = 0.15 (overall survival) for the comparison between cohorts in clinical stage III (log-rank test, 60-month analysis). (N)AT—Neoadjuvant and/or Adjuvant Treatment. Deaths and events shown reflect those occurring within the first five years (60 months) of follow-up, not the entire observation period. Within this window, no patient in the 2004–2013 cohort and 42 patients (10.2%) in the 2014–2023 cohort had less than 60 months of follow-up for overall survival and were censored. Over the complete follow-up, the total number of deaths was 323 in the 2004–2013 cohort and 163 in the 2014–2023 cohort.

**Table 4:** Multivariable Cox proportional hazards models for overall survival (OS) and cancer-specific survival (CSS) in stage I–III rectal cancer (n = 839; 60-month analysis).

Variable	HR	95% CI	*p*	HR	95% CI	*p*
	Overall Survival (OS)			Cancer-Specific Survival (CSS)		
Female sex	1.023	0.797–1.313	0.858	1.090	0.820–1.449	0.551
Age ≥ 65 years	2.250	1.671–3.029	<0.001	2.029	1.457–2.826	<0.001
Cohort period (2014–2023 vs. 2004–2013)	0.837	0.636–1.101	0.202	0.618	0.450–0.849	0.003
Mucinous/signet ring cell carcinoma	1.465	0.956–2.244	0.079	1.445	0.883–2.366	0.143
Tumour grade 2 (ref. grade 1)	0.911	0.675–1.228	0.539	0.866	0.615–1.221	0.413
Tumour grade 3 (ref. grade 1)	1.903	1.220–2.970	0.005	1.442	0.836–2.487	0.189
Mid rectal (ref. low rectal)	0.835	0.628–1.112	0.217	0.902	0.651–1.248	0.532
High rectal (ref. low rectal)	1.044	0.728–1.495	0.816	1.141	0.754–1.726	0.532
Clinical stage III (ref. stage I–II)	1.929	1.349–2.758	<0.001	2.035	1.310–3.161	0.002
Neoadjuvant ± adjuvant treatment	0.842	0.622–1.140	0.266	1.197	0.843–1.699	0.314
Adjuvant treatment only	0.979	0.691–1.388	0.907	1.050	0.691–1.597	0.818

## Data Availability

The data presented in this study are available on request from the corresponding author due to privacy and data protection requirements under Lithuanian law.

## Supplementary Materials

The following supporting information can be downloaded at: https://www.mdpi.com/article/10.3390/medicina62101888/s1, Figure S1: Participant flow diagram; Table S1: Timeline of clinical and diagnostic practice changes relevant to rectal cancer management.

## Abbreviations

The following abbreviations are used in this manuscript:

CI	Confidence Interval
CSS	Cancer-Specific Survival
FOLFOX	Folinic acid, Fluorouracil, Oxaliplatin
HR	Hazard Ratio
IQR	Interquartile Range
LUHS	Lithuanian University of Health Sciences
MDT	Multidisciplinary Team
MRI	Magnetic Resonance Imaging
NR	Not Reached
OS	Overall Survival
pCR	Pathological Complete Response
SEER	Surveillance, Epidemiology, and End Results
STROBE	Strengthening the Reporting of Observational Studies in Epidemiology
TME	Total Mesorectal Excision
TNT	Total Neoadjuvant Treatment

## References

[1] Sung H., Ferlay J., Siegel R.L., Laversanne M., Soerjomataram I., Jemal A., Bray F. (2021). Global Cancer Statistics 2020: GLOBOCAN Estimates of Incidence and Mortality Worldwide for 36 Cancers in 185 Countries. CA Cancer J. Clin..

[2] Dyba T., Randi G., Bray F., Martos C., Sanz A.S., Bettio M., Giusti F., Innos K., Ferlay J., Dimitrova N. (2021). The European cancer burden in 2020: Incidence and mortality estimates for 40 countries and 25 major cancers. Eur. J. Cancer.

[3] Glynne-Jones R., Moran B., Saunders M., Wyrwicz L., Tiret E., Brown G., Rödel C., Cervantes A., Arnold D., ESMO Guidelines Committee (2017). Rectal cancer: ESMO–ESSO–ESTRO Clinical Practice Guidelines for diagnosis, treatment and follow-up. Ann. Oncol..

[4] Jiang Y., Yuan H., Li Z., Chen M. (2021). Global pattern and trends of colorectal cancer survival: A systematic review of population-based registration data. Cancer Biol. Med..

[5] Allemani C., Matsuda T., Di Carlo V., Harewood R., Matz M., Nikšić M., Bonaventure A., Valkov M., Johnson C.J., Esteve J. (2018). Global surveillance of trends in cancer survival 2000–14 (CONCORD-3). Lancet.

[6] Cardoso R., Guo F., Heisser T., Hackl M., Innos K., Kokk R., Bórras J.M., Ortiz M.R.P., Bouvier A.M., Launoy G. (2022). Overall and stage-specific survival of patients with screen-detected colorectal cancer in European countries: A population-based study in 9 countries. Lancet Reg. Health Eur..

[7] Keller D.S., Berho M., Perez R.O., Wexner S.D., Chand M. (2020). The multidisciplinary management of rectal cancer. Nat. Rev. Gastroenterol. Hepatol..

[8] Aibuedefe B., Hamilton K.S., Yong V., Alimi Y., Obiri-Yeboah D., Jeyarajah D.R. (2022). It’s Not About the Money: Continued Treatment and Survival Outcome Disparities in Minority Rectal Cancer Patients After Controlling for Socioeconomic Factors. Ann. Surg. Oncol..

[9] Emile S.H., Horesh N., Freund M.R., Garoufalia Z., Gefen R., Silva-Alvarenga E., Bhatt H., Wexner S.D. (2023). Trends in the Characteristics, Treatment, and Outcomes of Rectal Adenocarcinoma in the US From 2004 to 2019: A National Cancer Database Analysis. JAMA Oncol..

[10] Ministry of Health of Lithuania (2009). Order No. V-508 on the Approval of the Financing Programme for Early Diagnosis of Colorectal Cancer. https://www.e-tar.lt/portal/lt/legalAct/TAR.18E44723B39F.

[11] Sauer R., Becker H., Hohenberger W., Rödel C., Wittekind C., Fietkau R., Martus P., Tschmelitsch J., Hager E., Hess C.F. (2004). Preoperative versus postoperative chemoradiotherapy for rectal cancer. N. Engl. J. Med..

[12] Hong Y.S., Nam B.H., Kim K.P., Kim J.E., Park S.J., Park Y.S., Park J.O., Kim S.Y., Kim T.W., Kang G.H. (2014). Oxaliplatin, fluorouracil, and leucovorin versus fluorouracil and leucovorin as adjuvant chemotherapy for locally advanced rectal cancer after preoperative chemoradiotherapy (ADORE). Lancet Oncol..

[13] Ministry of Health of Lithuania (2018). Order No. V-658 “On the Description of the Procedure for the Provision of Specialized Colorectal Cancer Treatment Services”. https://www.e-tar.lt/portal/lt/legalAct/f05e85f06b1211e8bbc2876081bf7fb5.

[14] Bahadoer R.R., Dijkstra E.A., van Etten B., Marijnen C.A.M., Putter H., Kranenbarg E.M., Roodvoets A.G.H., Nagtegaal I.D., Beets-Tan R.G.H., Blomqvist L.K. (2021). Short-course radiotherapy followed by chemotherapy before total mesorectal excision (RAPIDO): A randomised, open-label, phase 3 trial. Lancet Oncol..

[15] Conroy T., Bosset J.F., Etienne P.L., Rio E., François É., Mesgouez-Nebout N., Vendrely V., Artignan X., Bouche O., Gargot D. (2021). Neoadjuvant chemotherapy with FOLFIRINOX and preoperative chemoradiotherapy for patients with locally advanced rectal cancer (PRODIGE 23). Lancet Oncol..

[16] Valentini V., Alfieri S., Coco C., Di Giorgio A., De Quarto G. (2024). Four steps in the evolution of rectal cancer managements through 40 years of clinical practice: Pioneering, standardization, challenges and personalization. Radiother. Oncol..

[17] Piringer G., Ponholzer F., Thaler J., Kapp K., Augustin F. (2024). Prediction of survival after neoadjuvant therapy in locally advanced rectal cancer: A retrospective analysis. Front. Oncol..

[18] Li B., Han J., Wang F., Zhao Y., Shi Y., Xie W., Zhang Z. (2025). Factors affecting survival prognosis of patients with rectal cancer after neoadjuvant chemoradiotherapy. Front. Oncol..

[19] Park J.S., Park S.Y., Kim H.J., Cho S.H., Kwak S.G., Choi G.S. (2019). Long-term Oncologic Outcomes After Neoadjuvant Chemoradiation Followed by Intersphincteric Resection With Coloanal Anastomosis for Locally Advanced Low Rectal Cancer. Dis. Colon Rectum.

[20] Kim S., Huh J.W., Lee W.Y., Yun S.H., Kim H.C., Cho Y.B., Park Y.A., Shin J.K. (2024). Predicting survival in locally advanced rectal cancer with effective chemoradiotherapy response. Eur. J. Surg. Oncol..

[21] Wang S.J., Fuller C.D., Emery R., Thomas C.R. (2007). Conditional Survival in Rectal Cancer: A SEER Database Analysis. Gastrointest. Cancer Res..

