# Supplementary material for: Twenty Years of Modern Rectal Cancer Care in Lithuania: Stage Migration, Evolving Treatment Patterns, and Improved Survival at a Tertiary Referral Centre
Source: Medicina (Kaunas). 2026 Sep 29;62(10):1888. doi: 10.3390/medicina62101888 (PMC13707151; doi:10.3390/medicina62101888)
Supplement: Supplementary file 1 [file medicina-62-01888-s001.zip › medicina-4476163-supplementary.pdf]

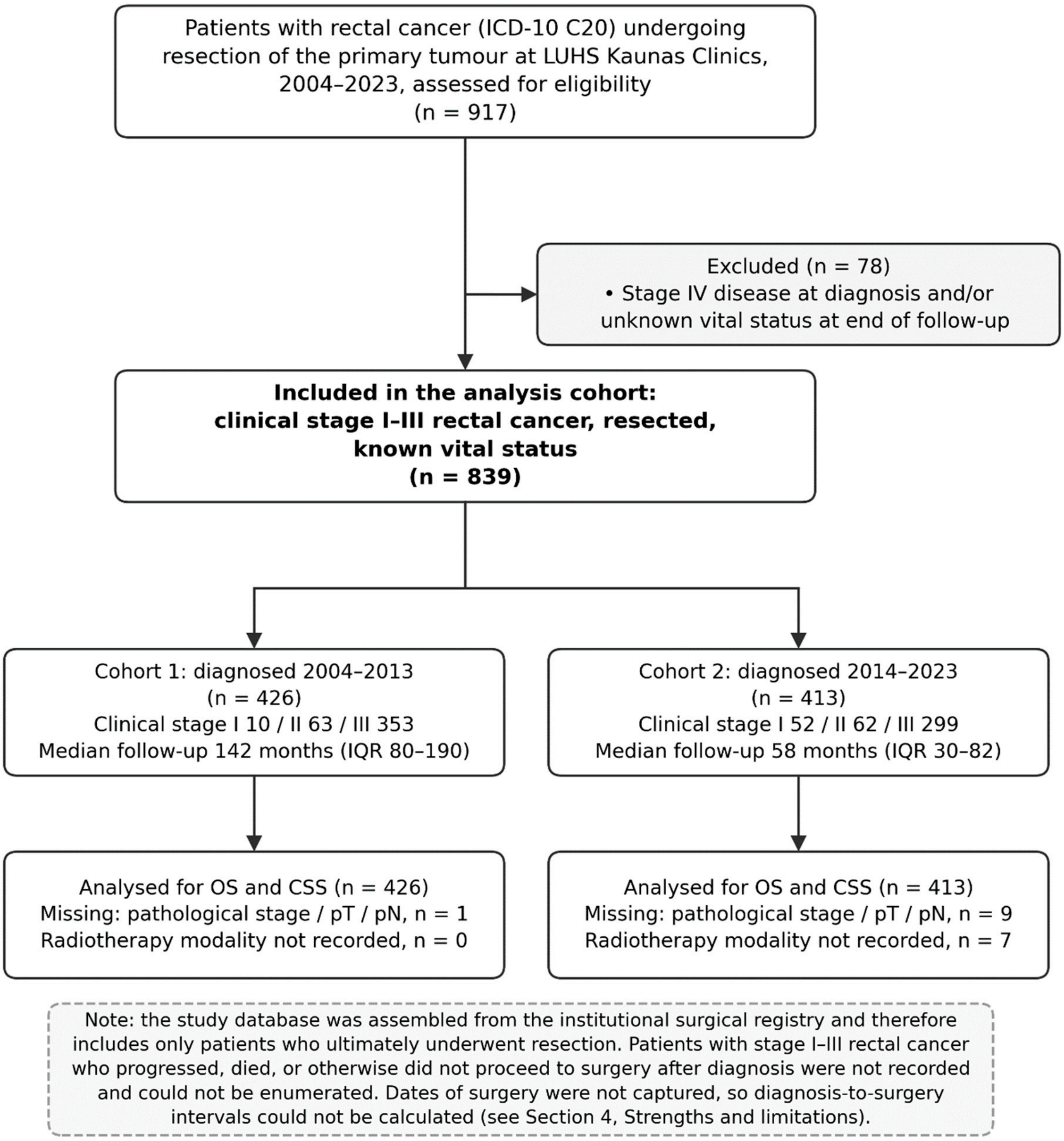

Figure S1. Participant flow diagram.

Table S1. Timeline of clinical and diagnostic practice changes relevant to rectal cancer management

Implementation Year	Diagnostic or Clinical Advancement	Possible Contribution to the Observed Trends
2002	Endorectal ultrasound (ERUS)	May have improved local T-staging and nodal assessment in early-stage rectal tumours and supported the selection of patients for local excision.
2004-2005	Standardization of Total Mesorectal Excision (TME)	Reported in the literature to reduce local recurrence through dissection within the mesorectal fascial plane with preservation of the pelvic autonomic nerves; local recurrence was not recorded in the present study.
2006	Introduction of neoadjuvant chemoradiotherapy	Standardised preoperative treatment following the CAO/ARO/AIO-94 trial, in which preoperative chemoradiotherapy was associated with lower toxicity and fewer local recurrences [11].
2008	Pelvic magnetic resonance Imaging (MRI)	Standardised preoperative local staging and assessment of the circumferential resection margin (CRM). This may have influenced treatment selection and also the recorded distribution of tumour location, as discussed in Strengths and limitations.
2013	Multidisciplinary Team (MDT) meetings on regular basis	Introduced a formalised, evidence-based approach to treatment planning that may have improved the selection and sequencing of multimodal therapy.
2014	National colorectal cancer screening program	May have contributed to the observed increase in earlier-stage diagnoses; its individual contribution to survival could not be quantified in this study.
2016	Routine chest, abdomen, and pelvic CT	May have improved detection of occult distant metastases and hence the accuracy of staging; this also affects the composition of the stage I–III cohort over time.
2018	Introduction of total neoadjuvant therapy (TNT)	In randomised trials TNT has been associated with higher pathological complete response rates and improved disease-free survival in locally advanced disease. In the present cohort the pathological complete response rate among patients treated with neoadjuvant chemoradiotherapy or TNT did not differ between eras (10.8% vs. 10.7%; p=0.98), so any contribution is more likely to reflect wider delivery of preoperative therapy than a change in per-patient tumour responsiveness.

2019	High-volume hospital designation	Consolidation of surgical expertise and multidisciplinary team experience; higher hospital volume has been associated with lower postoperative mortality in other settings, although this was not assessed in the present study

The entries in the right-hand column are possible contributions, based on published evidence and on the chronology of implementation at this institution. None of them was evaluated individually in this study, and the observed survival trends cannot be attributed to any single development.

Abbreviations: CRM, circumferential resection margin; CT, computed tomography; ERUS, endorectal ultrasound; LUHS, Lithuanian University of Health Sciences, Kaunas Clinics; MDT, multidisciplinary team; MRI, magnetic resonance imaging; (N)AT, (neo)adjuvant therapy; TME, total mesorectal excision.
